# Exploring the ethnic diversity of UK dentistry

**DOI:** 10.15694/mep.2018.0000055.1

**Published:** 2018-03-07

**Authors:** Patricia Neville

**Affiliations:** 1 University of Bristol

**Keywords:** ethnicity, dentistry, leaky pipeline

## Abstract

This article was migrated. The article was marked as recommended.

It is widely acknowledged that there are many social benefits to having a multicultural health force, including ensuring that there is less of a barrier around access for underrepresented groups. Despite this, certain healthcare professions, including dentistry, have struggled with the historical legacy of being conceptualised as a “white” profession and whether it is ethnically representative of the public it serves. This article examines the role and place of ethnicity in the dental profession in the UK. It will describe the changing ethnic composition of UK dentistry and highlight some of the challenges faced by the profession from an equality and diversity perspective. The current qualitative work of the author exploring the barriers and facilitators faced by students from an ethnic background pursuing a career in dentistry will also be introduced. It concludes by encouraging more research on this topic.

## Introduction

Dentistry, like other healthcare professions, has struggled with the historical legacy of being conceptualised as a “white” profession ( Adams, 1998). Related to this is the observation that dentistry is a privileged healthcare service, being more available to the populations of the developed North than those living in the developing South. Recent global dental workforce figures note that 69% of the world’s 1.6 million dentists only serve 27% of the world’s population because they are predominantly based in Europe and the Americas ( Gallagher and Hutchinson, 2018, p.1). The “inequitable distribution” ( Gallagher and Hutchinson, 2018, p.1) of dentists worldwide contributes to an oral health gap where the oral health needs at a population level are not met by local and national oral health services. In addition to this oral health inequity, the composition of the dental workforce also needs to be addressed. It is widely acknowledged that there are many social benefits to having a multicultural health force, including ensuring that there is less of a barrier around access for underrepresented groups (Smith et al., 2009, Nunn et al., 2014 all as cited in Sandino and Rowe 2014, p.465). This is because minority healthcare professionals tend to provide healthcare for the marginalised in society (Lacy et al., 2012, Smith et al., 2009, Onik, 2009 all as cited in Sandino and Rowe 2014, p.465). As a result, it is important that every country assesses the demographic composition of its dental workforce to ensure that it is ethnically representative of the public it must serve, and that dentistry is an equal opportunities profession. This article will present a review of the ethnic composition of UK dentistry and will ask the question: is dentistry in the UK ethnically diverse?

## Demographics: UK and UK dentistry

There are currently 65.6 million people living in the UK with 58.3 million living in England and Wales ( Office of National Statistics, 2017a). Census analysis of the ethnic composition of England and Wales reveals a predominantly white population of 48.2 million, accounting for 86% of the population. Those from an Indian ethnic background are the next largest ethnic group at 1.4 million or 2.5% of the population, followed by Pakistanis (2%). All other ethnic groups, including Black British are under 2% ( Office of National Statistics, 2012, p.5). Though these census figures portray a country that is predominately white, the proportion of this ethnic group has fallen from 91.3% in 2001 ( Office of National Statistics, 2012, p.4). Over the same period, a slight increase has been recorded in the numbers of Asian/Asian British, with those identifying as Pakistani increasing by 0.5% and Indians by 0.4% ( Office of National Statistics, 2012, p.6). The geographical spread of ethnicity reveals a distinctive pattern, with London being recorded as the most ethnically diverse area in England and Wales, with 50.29% of its population ethnically diverse. The West Midlands was the second most diverse region with 30.8% of the population from a minority ethnic group ( Office of National Statistics, 2012, p.7).

The clustering of ethnic diversity around key urban cities and the changes in the ethnic and cultural make-up of England and Wales has implications for the dental workforce. According to the Global Health Observatory Data Depositary (2015), the density of dentists in the UK (per 1000) has fallen from 1.549 in 1997 to 0.535, directly affecting the availability of dentists for the general population. In 2016, 41483 individuals were registered with the General Dental Council (GDC) to practice dentistry in the UK. Most of these registrants (72%) were UK qualified. 16% (6756) were EEA qualified, 7% (3112) qualified to practice in the UK by passing the ORE/UK overseas exam and 4% (1171) qualified overseas ( General Dental Council 2017, p.19). The place of qualification of registrants indicates that a proportion of practising dentists in the UK are international dentists, signalling a global workforce. Nevertheless, the largest proportion of UK dentists are domestically trained. In 2016, 49% of dentists registered with the GDC were “white” ( General Dental Council, 2017, p.25) and there is evidence that the profession is becoming more diverse. The next most significant ethnic identity is Asian or Asian British (19%), this is followed by Chinese or other ethnic background (3%), and Black British (1%). The fact that Asians are recorded as the largest non-white ethnic group in UK dentistry echoes their status as England and Wales’ second largest ethnic group ( Office for National Statistics, 2012, p.4).

The past decade has also seen an increase in the numbers of clinical academics from a Black, Asian and other Minority ethnic groups (BAME) background, rising from 14% in 2005 to 27.4% in 2016 ( Dental Schools Council, 2017, p.21). Nevertheless, only 26.8% of lecturers, 25.3% of senior clinical teachers and 28.9% of clinical teachers are from BAME backgrounds ( Dental Schools Council, 2017, p.21). Additionally, “BAME clinical academics are most underrepresented at Professor and Reader/Senior Lecturer grade (7.6% and 11.6% respectively), but also at Researcher level (where) they only make up 20% ( Dental Schools Council, 2017, p.21).”

Dental enrolment statistics offer us a glimpse into the future diversity of the profession. A review of the 2016/17 Higher Education Statistics Agency ( HESA 2017) data on the ethnicity of medical and dental students indicates a majority “white” composition (63.5%), followed by Asians (24.1%), Black (3.7%) groups. “Others” were recorded at 7.15% and 1.5% “unknown”. Based on these aggregates the medical and dental profession needs to work harder to improve its diversity at the level of student selection and admission. However, further examination of dental student enrolment data reveals more significant, albeit subtle changes across ethnic categories. Niven et al’s (2013, p.119) analysis of dental student enrolments based on the University and Colleges Admissions Service (UCAS) figures from 2007 and 2008 found that 46% of dental applicants were non-White; the largest group being that of Asian extraction ( Niven et al., 2013, p.119). Between 2010-2014 an average of 44.4% of British Asians applied for dentistry, with 37% being accepted to dental school. This compares with 41.6% of white students applying to dentistry and 52.2% being accepted into dental schools over the same time period ( Gallagher et al., 2017, p.184). Black British students are seriously underrepresented in UK dental schools (2%). Nevertheless, it is important to note that only 7% of students accepted to university in the UK are Black ( Gallagher et al., 2017, p.187).

## What is the issue?

The issue of widening participation is important for the future of the profession. The metaphor of the “pipeline” is often used to illustrate the challenges that under-represented groups (women, LGBTQ, ethnic minority groups) experience entering a profession or discipline that was traditionally closed to them (e.g. Berryman, 1983; Blickenstaff, 2005). This model proposes that the success of minorities groups in certain disciplines or professions is dependent on their ability to move through three key phases: first, gaining access into the profession/higher education; second, their successful participation in and completion of higher/professional education and third, how they progress into and develop their career ( Cronin and Roger, 1999). The above findings point to the existence of a “leaky pipeline” for students from BAME backgrounds who want to pursue a career in dentistry in the UK at both the point of educational access (college enrolments) as well as when they progress through their career and into specialist training. Additionally, this “leaky pipeline” does not appear to discriminate evenly- though Asian British are doing better that other minorities, the prospects for Black British in dentistry are particularly worrisome. The question remains: What are the barriers and facilitators to accessing, participating in and progressing in dentistry for BAME individuals?

Unfortunately, scholarship on this issue is limited in the UK. Though many researchers have investigated the challenges faced by non-traditional entrants into medicine, (e.g. Woolf et al., 2008; Woolf et al., 2011), less scholarly attention has been paid to uncovering the ethnic pipeline into dentistry. There have been some notable exceptions over the years, but these have been too sporadic to result in a comparable body of work. For instance, family pressure from Asian families for their children to pursue reputable and high-status professions, like dentistry, has been put forward to explain the representation of British Asians in dental student and occupational data via a vis other minority groups ( Lightbody et al., 1997). The positive impact that role models play in shaping peoples dental career has also been identified in the research ( Mohamed Osama and Gallagher, 2017). The high financial implications of studying dentistry have also been argued as a substantial barrier against the participation of Black British in dentistry ( Gallagher et al., 2017). Clearly, more research is needed to broaden our knowledge base on the barriers that people from BAME backgrounds encounter with aspirations to study dentistry. The over-representation of quantitative surveys and workforce analysis (see Bedi and Goldthorpe, 2000; Newton and Gibbons, 2001; Woolf et al., 2011; Dental Schools Council, 2017; Gallagher et al., 2017; Niven et al., 2013) has meant that the “voice” of minorities within the profession has been marginalised or forgotten. Adopting a mixed methods research methodology could generate novel information that will help us to support all students, but especially ethnic minority students, in professional education. It will also help redress a notable information gap in the literature and inform organisational change that would help institutions and the profession be more culturally sensitive.

Being a member of an ethnic minority group in a health force can be challenging. The 2017 publication of the UK’s National Health Service’s (NHS) NHS Workforce Race Equality Standard (WRES) (2016) recorded that while almost one in four NHS staff employed by the NHS are from a Black and Minority Ethnicity background (BME) ( WRES, 2017, p.6) (With 1.621 million employed by the NHS as of June 2017 ( Office of National Statistics, 2017b) that is the equivalent of 405,250 people), they were found to be significantly more likely to be disciplined by white staff members, and experience harassment, bullying and discrimination at work from colleagues and managers compared to their white colleagues ( WRES, 2017, p.11). As worrying as these findings are, it prompts the question whether BAME dentists experience “everyday racism” (Essed, 1991 as cited in Beagan, 2003, p.861) in their everyday encounters with patients and colleagues or institutional racism within dental hospitals. Research has yet to be conducted to shed light on this organisational aspect of UK dentistry.

## Future work

The above review of the field reveals the current gaps in the literature and signals the types of future work needed in this area. Further quantitative and qualitative research is required to explore the ‘known’ and ‘unknown’ challenges faced by people from BAME background at the point of educational access into the profession and as they progress through the profession. There is a predominance of quantitative studies measuring the ethnic composition of dental school enrolments and the ethnic composition of the dental workforce. Such studies allow us to measure the scale of ethnicity diversity of UK dentistry and identify areas for improvement or points of discussion. This quantitative data can in turn allow us to chart current and project future dental workforce demographics, aiding policy development and guidelines. Nevertheless, the proliferation of quantitative research is at the expense of producing participant-rich interpretative data on the experience of how identifying as white or BAME interjects, complicates or neutralises the professional development and trajectory of dentists. Having such first-person experiences could prove useful to support the design and development of evidence-based, participant centred programmatic supports for diversity and inclusivity initiatives within dentistry.

My own research interest lies with exploring dental students experiences of dental school. I am currently conducting a small scale qualitative study, interviewing a sample of dental students from a variety of ethnic backgrounds, on their access into and participation with dental school. It aims to explore how their ethnicity has influenced or shaped their interaction(s) with this profession and the educational institution that produces and reproduces future dentists. It has been undertaken to generate some much-needed qualitative data on the experiences of BAME students in dentistry in the UK. The lack of student centred research in dental education has been identified as a systemic problem by the profession ( Divaris et al., 2008; Henzi et al., 2007). There is also a notable absence of the experiences of minority students in dental education in the UK. This project is at the data collection stage, but if it proves successful, I plan to roll-out this project to other sites and increase the number of participants in the project.

I look forward to reading the contributions of the other authors of this Themed issue on diversity in medical education, sharing insights from their experience of working on this topic as well as learning from their research. I hope that my article will encourage further conservation about ethnic diversity within the dental profession in the UK.

## Take Home Messages

• Current statistics confirm the lack of ethnic diversity in UK dentistry. This is problem for the profession from an equality and diversity perspective, especially for those from a Black British background.
• Most research conducted on this issue uses quantitative methods. This research approach is helpful from a monitoring perspective.
• However, we have significant gaps in our knowledge about why certain ethnic groups are attracted to dentistry and others are not, and what are the challenges they encounter along the way.
• This article calls for more mixed methods research on the barriers and facilitators that face ethnic minorities who choose a career in dentistry.

## Notes On Contributors

Dr Patricia Neville is a Lecturer of Social Sciences at Bristol Dental School. She is Theme Lead for Personal and Professional Development (PPD) stream of the undergraduate BDS programme. Her teaching and research interests include: sociology of health, dental education, gender and ethnicity issues in dentistry and professionalism.
