# Supplementary material for: Exploring the ethnic diversity of UK dentistry
Source: MedEdPublish (2016). 2018 Mar 7;7:55. [Version 1] doi: 10.15694/mep.2018.0000055.1 (PMC10711999; doi:10.15694/mep.2018.0000055.1)
Supplement: Peer review history

# Reviewer report: Reviewer response for version 1

**DOI:** 10.21956/mep.19523.r28891

**Published:** 2018-03-26

**Authors:** Janusz Janczukowicz

**Affiliations:** 1 Medical University of Lodz

This review has been migrated. The reviewer awarded 4 stars out of 5 This is perfect to see the diversity of submissions to this special edition of MedEdPublish, not only form the perspective of the suggested framework but also the diversity of health and educational professions and authors ranging from undergraduate students to national organisations. This paper draws our attention to the ethnic diversity of UK dentistry and to barriers to widening participation in the dental profession. As suggested by the author, this introductory review should be followed by the research projects aimed at better understanding of complex interactions between the diversity-related barriers and challenges.

Reviewer Expertise:

NA

No decision status is available

# Reviewer report: Reviewer response for version 1

**DOI:** 10.21956/mep.19523.r28890

**Published:** 2018-03-09

**Authors:** Petra Verdonk

**Affiliations:** 1 VU University Medical Center, dpt Medical Humanities

This review has been migrated. The reviewer awarded 4 stars out of 5 The author addresses the issue of widening participation or fixing the numbers in UK dentistry, and she reveals the current gaps in literature and signals what type of work is needed in this area. Fixing the numbers is not only an issue of social justice in itself, but studies also show the benefits of a representative health care workforce for addressing the health care needs of underrepresented groups in society, thereby also often facing the ‘hardest jobs’. Dentistry is not only a privileged health service, it is also a white privileged health profession, and predominantly based in Europe and the Americas, thus, unevenly distributed across the world. This results in an oral health gap or oral health inequity. In the UK, the largest group of dentists are trained in the UK itself, but still a substantial number of physicians are international and half of the dentists are registered as ‘white’, with Asians as the largest non-white ethnic group in UK dentistry. Accessing, participating, and progressing the dentistry profession shows a leaky pipeline for several groups but for Black British individuals in particular, but little is known about the challenges they face. In surveys, minority voices are often marginalised or forgotten, and therefore, Neville advocates for a qualitative approach to studying and learning from the experiences of those identifying as BAME individuals in dentistry. This paper is a nice overview of ethnic diversity within the dentistry profession. We look forward to hearing about the results of Neville’s qualitative study in dentistry.

Reviewer Expertise:

NA

No decision status is available
